# Ants’ semiochemicals deters *Philaenus spumarius* (Hemiptera: Aphrophoridae) activity on olive trees

**DOI:** 10.1093/jee/toaf250

**Published:** 2025-10-07

**Authors:** Stefania Smargiassi, Alberto Masoni, Federico Cappa, Marco Valerio Rossi Stacconi, Filippo Chisci, Paride Balzani, Filippo Frizzi, Giovanni Benelli, Enrico Palchetti, Angelo Canale, Giacomo Santini

**Affiliations:** Department of Biology, University of Florence, Firenze, Italy; Department of Biology, University of Florence, Firenze, Italy; Department of Biology, University of Florence, Firenze, Italy; Research and Innovation Centre and Technology Transfer Centre, Fondazione Edmund Mach, San Michele all’Adige (TN), Italy; Department of Biology, University of Florence, Firenze, Italy; South Bohemian Research Center of Aquaculture and Biodiversity of Hydrocenoses, Faculty of Fisheries and Protection of Waters, University of South Bohemia, Ceske Budejovice, Czech Republic; Department of Biology, University of Florence, Firenze, Italy; Department of Agriculture, Food and Environment, University of Pisa, Pisa, Italy; Department of Agriculture, Food, Environment and Forestry (DAGRI), University of Florence, Piazzale delle Cascine, Florence, Italy; Department of Agriculture, Food and Environment, University of Pisa, Pisa, Italy; Department of Biology, University of Florence, Firenze, Italy

**Keywords:** *Crematogaster scutellaris*, meadow spittlebug, *Xylella fastidiosa*, trait-mediated interactions

## Abstract

The meadow spittlebug, *Philaenus spumarius* (Hemiptera: Aphrophoridae) (L.), is the primary vector of the bacterium *Xylella fastidiosa* (Xanthomonadales: Xanthomonadaceae) (Wells et al.), which causes a severe vascular disease leading to significant economic losses in olive production in southern Italy. In this study, we investigated the deterrent effect of ant scent on the behavior and activity of *P. spumarius* on olive twigs. Using binary choice experiments, we found that the compounds released by *Crematogaster scutellaris* (Hymenoptera: Formicidae) (Olivier) ants significantly reduced the time spent by spittlebugs on the olive twigs. Chemical analysis of ant cuticular composition, and of ant-scented and control olive leaves identified a mixture of specific compounds, which include octadecanal, triacontane, nepetalactol, and pelargonic acid, presumably involved in this interaction. Our findings contribute to the understanding of trait-mediated indirect interactions in agroecosystems and suggest further studies focused on behavioral bioassay-based experiments to evaluate the potential role of ants’ chemical cues in *P. spumarius* control to preserve olive groves.

## Graphical abstract

**Graphical Abstract:**
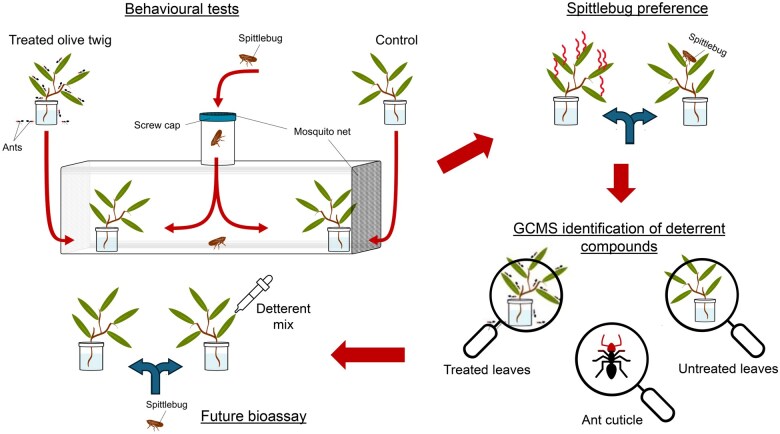

## Introduction

The meadow spittlebug *Philaenus spumarius* (L.) is a polyphagous xylem fluid-feeding species widespread in most terrestrial habitats worldwide (EFSA EFS 2016, Cornara et al. 2018). Information on its biology and ecology remains limited, largely because it was not previously regarded as an agricultural pest in Europe until the outbreak of the olive quick decline syndrome (OQDS) caused by *Xylella fastidiosa* (Wells et al.) (Xanthomonadales, Xanthomonadaceae) in Southern Italy more than 15 yr ago (Saponari et al. 2019). *P. spumarius* is the major vector of *X. fastidiosa*, the xylem-inhabiting bacterium agent of the OQDS, which is causing remarkable economic losses in olive production in Southern Italy (Cornara et al. 2017, Saponari et al. 2019). Estimates of future economic losses for Italy in the worst-case scenario range between 1.9 and 5.2 billion Euros, but this syndrome represents a severe threat to olive cultivation in the entire Mediterranean basin (Schneider et al. 2020, Benelli and Cornara 2021, Kottelenberg et al. 2021). The disease is characterized by significant damage to the olive tree’s vascular system, leading to leaf margin necrosis, progressive wilting and desiccation of leaves, often resulting in plant death (Cornara et al. 2019) and consequently in habitat biodiversity and landscape services loss (Valente et al. 2024). Currently, there are no effective and sustainable phytosanitary control measures against *X. fastidiosa* on infected plants. Thus, adopting agronomic practices is crucial to prevent plant infection and disease transmission (Morelli et al. 2021). Several strategies focus on reducing the insect vector populations of this bacterium (Cornara et al. 2018, 2025, Avosani et al. 2024, Bedini et al. 2025). The removal of ground cover, for example, is a common strategy to reduce populations of *P. spumarius*. However, although this technique may reduce the number of immature instars (ie, nymphs), the contrast in the color between the plants and the surrounding soil is likely to attract adult individuals (Avosani et al. 2024). An alternative management approach that could reduce the presence of *P. spumarius* individuals could be the exploitation of trait-mediated indirect interactions (TMIIs), where parasites alter their behavior in response to specific cues (eg, pheromones) to avoid predation risk, even in the absence of the predator itself (Ohgushi et al. 2012).

It is well known that communication among ants occurs by releasing chemical substances used in all major social activities (Hölldobler and Wilson 1990, David Morgan 2009, Angulo et al. 2024). Ants emit a wide range of semiochemicals for intra- and interspecific communication, eliciting behavioral or physiological responses in recipients (Hölldobler 1978, Attygalle and Morgan 1984). However, the perception of these compounds can also be exploited by organisms that are not the primary targets of communication, deriving benefits from them (Adams et al. 2020). For instance, the ant-eating spiders, that is *Habronestes bradleyi* (O. P-Cambridge) (Araneae, Zodariidae) or *Habrocestum pulex* (Hentz) (Araneae, Salticidae) rely on alarm pheromones released by ants to locate and prey on them (Allan et al. 1997, Clark et al. 2000). Many different types of ant pheromones used in intra- and interspecific communication could also be detected by their potential prey, raising avoiding behavior (Jackson and Ratnieks 2006, Van Mele 2008, Offenberg 2015). Recent studies have shown that pest species such as the fruit flies *Ceratitis capitata* (Wiedemann) (Diptera, Tephritidae), *Ceratitis cosyra* (Walker) (Diptera, Tephritidae), and *Bactrocera invadens* (Drew-Tsuruta and White) (Diptera, Tephritidae) alter their behavior, reducing the time spent on fruits and avoiding oviposition where ant presence has been detected (Van Mele et al. 2009, Smargiassi et al. 2023). In addition, it has been seen that the African weaver ants *Oecophylla longinoda* (Latreille) (Hymenoptera, Formicidae) can be used as vectors of entomopathogenic fungi to enhance the biological control of tephritid fruit fly pests. The ants can spread these fungi among pest populations, increasing the mortality of the target pests (Nève de Mévergnies et al. 2025). A study conducted on the invasive ragweed beetle *Xylosandrus compactus* (Eichhoff) (Coleoptera, Curculionidae) also showed a clear preference of choice for twigs of *Castanea sativa* (Miller) (Fagales, Fagaceae) and *Laurus nobilis* (L.) (Laurales, Lauraceae) that had not been in contact with ants, compared to those previously exposed to 4 different ant species (Giannetti et al. 2022). Therefore, TMIIs represent important mechanisms through which ants influence other species and alter ecosystem dynamics (Bolker et al. 2003, Werner and Peacor 2003), with potential applicability to the management of pest species (Verheggen et al. 2010, Offenberg 2015).

Given that ants can be potential predators of the spittlebugs (Sujii et al. 2002, Nachappa et al. 2006), especially in the juvenile stages (Balzani et al. 2023), in this study, we investigated whether substrates bearing chemical compounds released by the Mediterranean acrobat ant *Crematogaster scutellaris* (Olivier) (Hymenoptera, Formicidae) are avoided by *P. spumarius*.

*C. scutellaris* is a tree-nesting myrmicine ant species widely distributed across natural and human-managed Mediterranean ecosystems, including olive orchards (Santini et al. 2007). These ants typically establish large colonies, comprising thousands of individuals, exhibiting monogynous and polydomous nesting behaviors (Frizzi et al. 2020, Masoni et al. 2020). Furthermore, this ant species is known to prey on various insect pests, including *P. spumarius* (eg, Balzani et al. 2023). Herein, we performed behavioral assays (ie, binary choice tests) where spittlebugs could freely choose between olive twigs that had not been previously in contact with ants and those on which ants were allowed to walk before the experiment. We then assessed whether there was a perception of potential ant-borne cues and the subsequent avoidance response. Considering the persistence of such cues (Van Mele et al. 2009, Smargiassi et al. 2023), we conducted a chemical analysis of the substrates to identify the compounds involved in this interaction and then further compared them with the cuticular profile of *C. scutellaris* ants.

## Materials and Methods

### Study Sampling and Setup

Fourth and fifth instar nymphs of *P. spumarius* were collected from the field inside the Florence University Science Campus (43°49′00″ N, 11°11′59″ E) in April 2022 and placed in a greenhouse, on pot-growing pea plants (*Pisum sativum* (L.) (Fabales, Fabacee) cv. dwarf pea Rainer, Blumen, Italy) covered by mesh cages, until adult emergence. Adults were individually reared on single sunflowers (*Helianthus annuus* (L.) (Asterales, Asteraceae) cv. Giant sunflower, Blumen, Italy) seedlings for a week inside a climatic chamber (25 ± 0.5 °C, L16: D8, RH 75 ± 5%) before being employed in the experiments. *C. scutellaris* workers were collected from colonies inhabiting oak trees in the campus area a few days before the experiments. The trees have not been treated with pesticides, at least in the last 10 yr. Ants were housed in plastic containers (15 × 23 × 15 cm, about 300 workers each) previously coated with Fluon (AGC Chemicals Europe, Ltd) to prevent ants’ escape and maintained in a climatic chamber under the same conditions as the adult spittlebugs, providing sugar solution and water ad libitum.

**Fig. 1.**
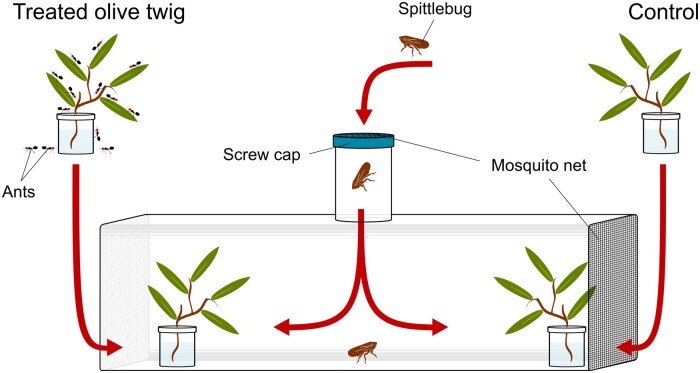
Set up of the experimental arena and of the test trials.

One hundred twigs of the current year with 7 leaves were cut from 20-yr-old olive trees *Olea europaea* (L.) (Scrophulariales: Oleaceae) (cv. Frantoio) grown as ornamental trees and not subjected to any agronomic treatment except pruning. Each twig was individually placed in a plastic jar (5 cm diameter and 8 cm high) with a perforated cap containing tap water to prevent desiccation (Fig. 1). The insertion point of the twig in the jar was sealed with Parafilm (Bemis Company, Sheboygan Falls, Wisconsin, USA) to prevent ants from entering the jar (Fig. 1). Half of the jars were then placed into glass cages (50 × 30 × 20 cm, 10 twigs per box) together with 600 ant workers, which were free to move on the twigs. The other half of the twig-carrying jars (controls) were inserted in empty containers and were therefore not exposed to ants. After 48 h, the jars were removed from the containers using sterile nitrile gloves and avoiding touching the twigs. The treated twigs were carefully removed from the glass cages when no more ants were walking on them and without harassing the ants. We also checked that the leaves had not been damaged by the ants.

### Behavioral Assay

One treated and one control twig were placed at the opposite ends of each experimental arena (*n* = 10, Fig. 1). The left and right sides of each arena were randomly alternated to avoid any spatial bias. The arenas were cylindrical chambers (diameter: 25 cm, length: 70 cm) with transparent chiffon fabric at both ends to allow free air influx. An adult spittlebug was gently placed into the arena through a central inlet (Fig. 1) and observed for 10 min every hour for a total of 8 h, starting at 10 AM. The arenas were ideally divided into 3 equally sized (23 cm) parts: the treatment side, a central “no choice” area, and the control side. At each time interval, we recorded the position of the spittlebug (ie, treatment, central, and control side) and whether it stayed on the twig or the arena. The test was repeated 40 times with different adult spittlebugs and new twigs each time.

The probability of a spittlebug moving to the treatment or control compartment was modeled using generalized linear mixed models (GLMM) with binomial distribution using the time (hours) as a predictor. Arena identity was used as a random term, to account for repeated observations. The significance of terms was assessed using type II analysis of variance with the Wald chi-square test. All analyses were performed using version 4.0.3 of the R software (R Core Team 2020), with the packages “lme4” (Bates et al. 2015) and “car” (Fox et al. 2001).

### Chemical Analysis

Compound extractions were performed on 20 treated (ant-scented) and 20 untreated (negative control) olive leaves. Each leaf was extracted in a glass vial with 3 ml of heptane for 60 s. Heptane was used as solvent for extraction due to its lower evaporation rate compared to pentane or hexane, to minimize changes in the solvent volume during the 60 s of extraction. As a positive control, we also extracted the apolar fraction of the cuticular profile from ants. Each sample (*n* = 10) was a pool of 10 freeze-killed ants collected from the treated leaves. Extraction was carried out by placing the pooled ants in a glass vial with 1 ml of heptane for 10 min (Sprenger et al. 2020). All the extracts were then evaporated at room temperature and re-dissolved in 20 μl of heptane containing n-hexadecanol (n-C16OH) as an internal standard at a concentration of 40 ng μl^−1^ to correct for extraction efficiency and injection volume variability.

For compound identification, 1 μl of each resuspended extract was injected in splitless mode into a Hewlett-Packard (Palo Alto, California, USA) 7820A gas chromatograph (GC) coupled to an HP 5977B mass selective detector (using a 70-eV electronic ionization source). A fused ZB-WAX-PLUS (Zebron) silica capillary column (60 m × 0.25 mm × 0.25 mm) was installed in the GC. The injector port and transfer line temperatures were set at 200 °C and the carrier gas was He (at 20 PSI head pressure). The run time of each sample was 48 min. The oven temperature was programmed to increase from 70 to 150 °C at 30 °C min^−1^ and from 150 to 320 °C at a rate of 10 °C min^−1^, with the final temperature held for 5 min. The injector port and transfer line temperatures were set at 200 °C and the carrier gas was He (at 15 PSI head pressure). Injections were performed in splitless mode (1min purge valve off). The output was recorded with the Agilent Mass Hunter Workstation program and the data acquisition was done using the Chem Station G1701 BA software (v. B.01.00 Hewlett-Packard).

Compound identification was based on comparisons of mass spectra and calculated retention indices with entries in the Wiley mass spectra database (Wiley Registry of Mass Spectra Data 2022 edition, Hoboken, New Jersey, USA). No synthetic standards were run in the laboratory for identification. Quantification of the detected compounds was performed by integrating each analyte’s peak area and normalizing it to the peak area of the internal standard (n-hexadecanol at 40 ng μl^−1^) added to each extract prior to analysis. This provided relative abundance ratios (analyte peak area/internal standard peak area), which were averaged across replicates for each treatment and reported as mean ± standard deviation.

Because synthetic standards for the detected compounds were not available, absolute quantification was not performed. Instead, data were used for comparative analyses between treatments. The limit of detection (LOD) and limit of quantification (LOQ) were calculated for each replicate injection based on the signal-to-noise ratio (S/N). The LOD, defined as 3 times the S/N, averaged approximately 0.6 ng per injection, while the LOQ, defined as 10 times the S/N, averaged approximately 2 ng per injection, reflecting the instrument sensitivity under the acquisition parameters used.

## Results

### Behavioral Assay

**Fig. 2.**
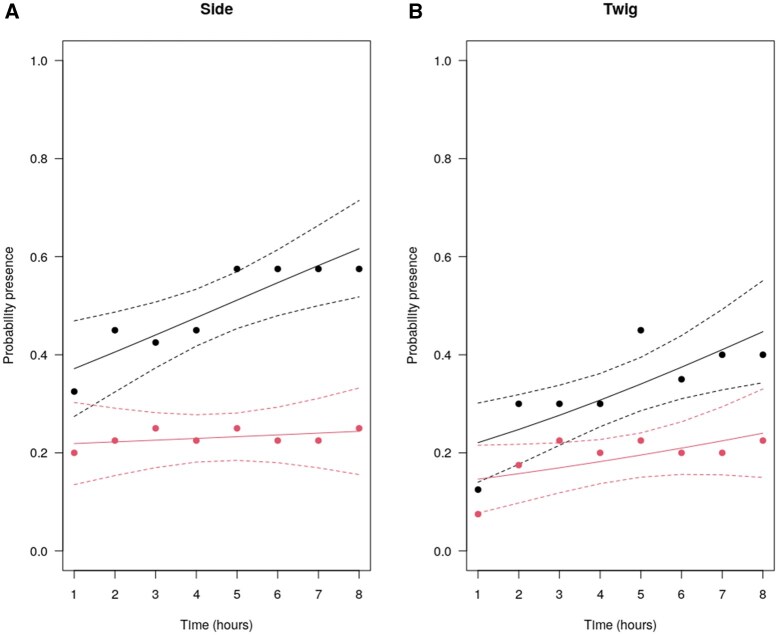
Probability of *Philaenus spumarius* presence on controls (black dots) or treatments (red dots) of A) side of the experimental chamber and B) olive twigs. Dots: observed frequencies; continuous lines: predicted values from GLMM; dashed lines: 95% confidence intervals.

*P. spumarius* probability of moving to the control side of the chamber was significantly higher than the probability of moving to the treatment side (Fig. 2A). This probability increased through time (χ^2^ = 26.71, df = 1, *P *< 0.0001) and reached its maximal value (∼0.6) after 5 h from the beginning of the experiment. On the contrary, the probability of occupying the treatment side of the chamber did not vary over time (χ^2^ = 0.24, df = 1, *P *= 0.62), remaining stable at around 0.2. A similar but less marked trend was observed when considering the spittlebugs that climbed upon a twig (Fig. 2B). Even in this case, the probability of choosing the control twig was higher than the opposite. When a spittlebug jumped on a twig, it rapidly moved on the leaf petiole or into the lower part of the leaf. All models’ results are shown in Online Resource 1.

### Chemical Analysis

Chemical analyses identified 39 compounds, 25 of which were consistently detected in the positive controls, 19 in the negative controls, and 22 in the treatment samples. These 39 compounds were represented by alkanes (*n*. 22), aldehydes (*n*. 10), carboxylic acids (*n*. 6), and monoterpenes (*n*. 1) (Table 1). For each substance detected, the peak area of the compound was calculated through integration and compared with the internal standard peak to obtain a quantitative result (Table 2). Octadecanal, triacontane, nepetalactol, and pelargonic acid were the only compounds detected in both treated leaves and positive controls (ant cuticular extracts), but not in negative controls (untreated olive leaves) (Table 1).

**Table 1.** Table of chemical compounds, including formulas, molecular weights (MW) and retention times (RT) for different groups

Compound	Formula	MW	Group	RT min	RT max	C	T	A
2,4-Nonadienal, (E, E)-	C9H14O	138.21	Aldehydes	7.247	7.253			7
2,4-Decadienal, (E, E)-	C10H16O	152.24	Aldehydes	8.329	8.335			4
Decanal	C10H20O	156.26	Aldehydes	7.144	7.156	3	10	
(7/11Z) Tetradecenal	C14H26O	210.36	Aldehydes	12.552	12.632			7
Tetradecanal	C14H28O	212.37	Aldehydes	12.786	12.872			10
Pentadecanal-	C15H30O	226.4	Aldehydes	14.909	14.915			6
cis, cis-7,10,-Hexadecadienal	C16H28O	236.39	Aldehydes	15.887	15.893			4
cis-9-Hexadecenal	C16H30O	238.41	Aldehydes	16.608	16.694			10
(9/13Z)-Octadecenal	C18H34O	266.5	Aldehydes	18.88	18.96			8
Octadecanal	C18H36O	268.5	Aldehydes	17.049	17.123		6	9
Tetradecane C14	C14H30	198.39	Alkanes	9.113	9.21	4	5	
Hexadecane C16	C16H34	226.44	Alkanes	12.523	12.54	11	16	
Heptadecane C17	C17H36	240.5	Alkanes	14.606	14.617	2	10	
Octadecane C18	C18H38	254.5	Alkanes	16.706	16.734	16	17	
Eicosane C20	C20H42	282.5	Alkanes	20.717	20.757	16	19	
Docosane C22	C22H46	310.6	Alkanes	24.402	24.413	16	11	
Tricosane C23	C23H48	324.6	Alkanes	26.13	26.141	7	8	
Tetracosane C24	C24H50	338.7	Alkanes	27.772	27.818	14	4	
Hexacosane C26	C26H54	366.7	Alkanes	30.891	30.982	16	10	10
Heptacosane C27	C27H56	380.7	Alkanes	32.39	32.636	9	13	9
Octacosane C28	C28H58	394.8	Alkanes	33.803	33.935	15	19	10
Nonacosane C29	C29H60	408.8	Alkanes	35.319	35.525	11	14	7
Triacontane C30	C30H62	422.48	Alkanes	36.017	36.063		2	10
Dotriacontane C32	C32H66	450.51	Alkanes	38.529	39.342	14	17	7
Tritriacontane C33	C33H68	464.9	Alkanes	40.509	40.778	11	15	
3-Tetradecene, (E)-	C14H28	196.37	Alkenes	9.061	9.107			8
1-Pentadecene	C15H30	210.4	Alkenes	10.406	10.469			10
1-Eicosene	C20H40	280.5	Alkenes	22.279	22.302	5	6	
Pelargonic acid	C9H18O2	158.24	Fatty acids	7.814	7.865	1	6	2
Palmitoleic acid	C16H30O2	254.41	Fatty acids	19.647	19.859			7
Palmitic acid	C16H32O2	256.42	Fatty acids	20.082	20.11			10
Linoleic acid	C18H32O2	280.4	Fatty acids	23.246	23.252			10
Oleic acid	C18H34O2	282.5	Fatty acids	23.441	23.595			10
Stearic acid	C18H36O2	284.5	Fatty acids	23.887	23.927			6
Farnesane	C15H32	212.41	Me-alkanes	10.023	10.034	6	3	
Tetradecane, 2,6,10-Trimethyl-	C17H36	240.5	Me-alkanes	10.561	10.572	3	7	
Octacosane, 2-methyl-	C29H60	408.8	Me-alkanes	37.311	37.351			8
Nepetalactol	C10H16O2	168.23	Monoterpenes	8.014	8.02		5	9

For each compound, the retention times are given with the corresponding minimum (RT min) and maximum (RT max) values. In addition, the presence of each compound in the positive control samples (group A—ant cuticular extracts), the treated samples (group T—treated olive leaves), and the negative control (group C—untreated olive leaves) is indicated.

**Table 2.** Relative abundance ratios (analyte peak area/internal standard peak area) of chemical compounds analyzed in ant samples from group A (ant cuticular extracts—positive controls), group T treated samples (treated olive leaves), and group C (untreated olive leaves—negative controls)

Relative abundance ratios	A		T		C	
	Mean	SD	Mean	SD	Mean	SD
(7/11Z) Tetradecenal	0.0088	0.0047				
(9/13Z)-Octadecenal	0.0072	0.0037				
1-Eicosene			0.0178	0.0048	0.0153	0.0034
1-Pentadecene	0.0110	0.0053				
2,4-Decadienal, (E, E)-	0.0040	0.0012				
2,4-Nonadienal, (E, E)-	0.0068	0.0021				
3-Tetradecene, (E)-	0.0038	0.0015				
cis, cis-7,10,-Hexadecadienal	0.0121	0.0023				
cis-9-Hexadecenal	0.0381	0.0166				
Decanal			0.0299	0.0351	0.0063	0.0042
Docosane C22			0.1044	0.0261	0.1363	0.0121
Dotriacontane C32	0.7448	0.1193	5.5507	1.3766	6.3341	0.8988
Eicosane C20			0.0863	0.0249	0.1247	0.0202
Farnesane			0.0081	0.0009	0.0097	0.0019
Heptacosane C27	4.3625	1.7109	1.5040	0.5973	1.2559	0.3931
Heptadecane C17			0.0190	0.0131	0.0216	0.0052
Hexacosane C26	0.1442	0.0551	0.1717	0.0503	0.2052	0.1072
Hexacosane, 9-Octyl-	0.4020	0.2842	0.3401	0.2257		
Hexadecane C16			0.0427	0.0316	0.0877	0.0743
Linoleic acid	0.0442	0.0447				
Nepetalactol	0.0050	0.0026	0.0104	0.0055		
Nonacosane C29	3.0880	1.4810	9.8342	5.5776	10.6692	3.8055
Octacosane C28	0.7676	0.2879	0.7943	0.2456	0.9805	0.3269
Octacosane, 2-methyl-	0.7945	0.1185				
Octadecanal	0.0189	0.0089	0.0164	0.0025		
Octadecane C18			0.1105	0.0422	0.1274	0.0211
Oleic acid	0.1822	0.1769				
Palmitic acid	0.0942	0.0934				
Palmitoleic acid	0.0614	0.0444				
Pelargonic acid	0.0050	0.0014	0.0206	0.0132	0.0220	#DIV/0!
Pentadecanal-	0.0057	0.0020				
Stearic acid	0.0540	0.0411				
Tetracosane C24			0.1071	0.0401	0.1637	0.0439
Tetradecanal	0.0072	0.0034				
Tetradecane C14			0.0154	0.0094	0.0472	0.0183
Tetradecane, 2,6,10-Trimethyl-			0.0078	0.0034	0.0124	0.0103
Triacontane C30	0.7278	0.0972	0.2200	0.0436		
Tricosane C23			0.0194	0.0050	0.0243	0.0118
Tritriacontane C33			0.4462	0.3168	0.3940	0.3314

For each compound, mean values and standard deviations (SD) are provided for each group, highlighting concentration variations between groups.

## Discussion

Our results suggest that the previous exposure of olive twigs to *C. scutellaris* ants triggers avoidance responses in adults of *P. spumarius*. Despite their limited olfactory ability (Avosani et al. 2024), spittlebugs perceived past ant presence in the treated twigs and avoided them.

The avoidance of ant-scented twigs remained for the entire duration of experimental observations (8 h after ant removal). Moreover, the number of spittlebugs choosing the control side in the test chamber gradually increased over time, as more individuals moved from the neutral zone towards the control side. Persistence times of semiochemicals described in different ant species can be highly variable; for example, for the garden black ant *Lasius niger* (L.) (Hymenoptera, Formicidae), the persistence of traces released upon their passage is 20 to 24 h (Evison et al. 2008), but in species such as *O. longinoda* it has been seen to be up to 9 wk (Beugnon and Déjean 1992). Smargiassi et al. (2023) showed that *C. capitata* avoidance of substrates previously visited by *C. scutellaris* persisted for at least 3 consecutive days after the removal of ants.

Chemical analyses revealed 4 interesting substances, namely octadecanal, triacontane, nepetalactol, and pelargonic acid, which were detected on both treated leaves and ant cuticular extracts, but not on untreated leaves used as negative controls. Previous research indicated that all these 4 compounds appear to have a repellent effect on several insect species or are essential components of products used as repellent agents (Naik et al. 2008, Zhu et al. 2012, Mukherjee 2015).

The presence of nepetalactol in our treated samples, as well as on ant cuticles, is of particular interest. Indeed, this monoterpene is also produced by plants such as catnip *Nepeta cataria* (L.) (Lamiales, Lamiaceae) and it is an effective insect repellent (McElvain et al. 1941, Ebrahim et al. 2015). Zhu et al. (2012) demonstrated that the application of catnip oil and its constituent compounds, nepetalactones, reduced the egglaying of the horse fly *Stomoxys calcitrans* (L.) (Diptera, Muscidae) by up to 98%. Similar effects were also found in *Drosophila melanogaster* (Meigen) (Diptera, Drosophilidae), whose larvae and adults can specifically detect nepetalactol and actinidine released by their parasitoid wasp *Leptopilina*, avoiding sites soaked with this substance (Ebrahim et al. 2015). Nepetalactone is also a component of aphids’ sex pheromone, and could potentially be released by ants, together with other compounds, to attract them (Stadler and Dixon 2005, Lu et al. 2024). Octadecanal belongs to the class of aldehydes, composed of a linear aliphatic chain of 18 carbon atoms with an aldehyde functional group (−CHO) at the end. In ants, octadecanal, along with the other compounds secreted by the metapleural gland, plays several roles, as in trail marking or deterring predators (Yek and Mueller 2011, Vieira et al. 2012). Additionally, octadecanal has also been found in the tarsal secretions of the bumblebees *Bombus* (Latreille) (Hymenoptera, Apidae) and, along with other substances, plays a crucial role in communication during the foraging of this species (Schmitt et al. 1991).

As for triacontane, its presence was found in only 2 of the treated samples, but it was detected in all the ant cuticular samples and never in the negative controls. The amount released and/or the persistence of this substance may therefore not be extremely high, although it could still contribute to the deterrent effect. Previous studies suggested that triacontane could also be one of the compounds emitted by some plants, like *Momordica cochinchinensis* (Spreng.) (Violales, Cucurbitaceae), which could affect the behavior of insects (Mukherjee 2015, Singh et al. 2019). However, as we never found this compound on untreated leaves, we can exclude that it was produced by olive twigs.

Nonanoic acid has been found in extracts of the gasters and thoraxes, in pheromone trails, in the Dufour’s or the mandibular gland of several ant species belonging to different genera (Huwyler et al. 1975, Fales et al. 1984, Attygalle et al. 1987, Hogan et al. 2017). The consistent presence of this compound in different species across several genera may suggest that it is a conserved trait with specific functions. Nonanoic acid is currently used as a component in some mosquito repellents, and it has also the characteristic of reducing egg-laying by these insects. Schultz et al. (1982) revealed that several aliphatic carboxylic acids can help prevent egg-laying and, among these, the one with the most promising effect was nonanoic acid. Nonanoic acid has also an antifeedant effect on adults of the large pine weevil, *Hylobius abietis* (L.) (Coleoptera, Curculionidae) (Månsson et al. 2006). The widely documented repellent properties of this substance are promising, and the possibility of its effective use against some pests may be concrete, potentially extending these benefits also to the treatment of olive trees.

In conclusion, this study has demonstrated that the release of certain chemical substances by a common Mediterranean ant species has a deterrent effect on *P. spumarius* in olive groves. The results suggest that spittlebugs can perceive the presence of predatory ants through specific chemical compounds released by the ants. Although it is unclear whether the presence of ants on the leaves also leads to the release of VOCs, the lasting nature of this repellent effect indicates that it is likely due to more persistent, less volatile compounds. Additionally, previous experiments with the same ant species on other substrates yielded the same repellent effect, even days after the exposure (Giannetti et al. 2022, Smargiassi et al. 2023). *P. spumarius* from olive twigs, highlighting a promising avenue for natural pest management in olive groves. The identification of key compounds (nepetalactol, octadecanal, triacontane, and nonanoic acid) supports the idea that ant-derived semiochemicals can exert long-lasting ecological effects on insect behavior. Despite the promising avenue for practical application of these results, it is important to clearly discuss some limitations of this study. In our research, we first described the repellent effect of previous ant presence and then we concluded that the semiochemicals produced by the ants trigger the escape response of *P. spumarius*. A final word on this issue can be given only with specific bioassays, where these compounds are applied to the plants, without any form of contact with ants. Such experiments will involve testing different concentrations of these molecules, either individually or in combination to check for synergistic effects and find the optimal combination. Finally, a deeper understanding of TMII between ants and herbivorous insects can certainly pave the way for innovative and eco-friendly pest management strategies based, for example, on stimulating ant presence on olive trees. With our study, we hope to stimulate further research on these issues.

## Data Availability

The datasets generated during and analyzed during the current study are available at doi: https://doi.org/10.6084/m9.figshare.21610815.v2
